# Molecular Mechanisms of Nutrient-Mediated Regulation of MicroRNAs in Pancreatic β-cells

**DOI:** 10.3389/fendo.2021.704824

**Published:** 2021-11-04

**Authors:** Anna Sałówka, Aida Martinez-Sanchez

**Affiliations:** Section of Cell Biology and Functional Genomics, Department of Metabolism, Digestion and Reproduction, Faculty of Medicine, Imperial College London, London, United Kingdom

**Keywords:** miRNA, islet, β-cell, type 2 diabetes, glucose, nutrients, diet

## Abstract

Pancreatic β-cells within the islets of Langerhans respond to rising blood glucose levels by secreting insulin that stimulates glucose uptake by peripheral tissues to maintain whole body energy homeostasis. To different extents, failure of β-cell function and/or β-cell loss contribute to the development of Type 1 and Type 2 diabetes. Chronically elevated glycaemia and high circulating free fatty acids, as often seen in obese diabetics, accelerate β-cell failure and the development of the disease. MiRNAs are essential for endocrine development and for mature pancreatic β-cell function and are dysregulated in diabetes. In this review, we summarize the different molecular mechanisms that control miRNA expression and function, including transcription, stability, posttranscriptional modifications, and interaction with RNA binding proteins and other non-coding RNAs. We also discuss which of these mechanisms are responsible for the nutrient-mediated regulation of the activity of β-cell miRNAs and identify some of the more important knowledge gaps in the field.

## Introduction

Over 8.5% of the world’s adult population suffer diabetes, which causes annually more than 1.5 million deaths (1). Type 1 diabetes (T1D) is a chronic autoimmune disease that usually leads to a severe loss of insulin-secreting pancreatic β-cells, whereas type 2 diabetes (T2D) is generally characterized by a reduced capability of peripheral tissues to respond to insulin and the incapacity of β-cells to secrete enough hormone to compensate for the higher demand. Both forms of the disease, if untreated, result in chronic hyperglycaemia that strongly contributes to the development of cardiovascular, neurological, kidney, and other complications. These result in significant morbidity, a strong reduction in life quality and expectancy, and colossal costs to health services (1).

Besides the relevance of loss in β-cell mass for the development of both T1D and, to a lesser extent, T2D (2), recent studies have suggested that impaired β-cell function plays a key role in early stages of both diseases (3). Whilst glucose metabolism in the β-cell is an important positive regulator of β-cell survival and regeneration (4), chronic hyperglycaemia impacts both β-cell function and survival (5). Adipose tissue secretes adipokines and sequesters fatty acids (FA) such as triglycerides to maintain glucose homeostasis (6). Obesity can cause adipose tissue dysfunction that contributes to peripheral insulin resistance (6). Furthermore, β-cell failure can result from the deleterious effects of high circulating free fatty acid levels, inflammation, and as recently proposed, pancreatic lipid deposition (7, 8).

Since their discovery less than 30 years ago, microRNAs (miRNAs) have been studied under each conceivable experimental condition and cellular context and are now well established as essential regulators of cellular function and important contributors to human disease (9). It was almost 20 years ago that the first islet-specific miRNA, miR-375, was identified and characterized by the Stoffel lab (10) and that Lynn et al. demonstrated that miRNAs are essential for the development of pancreatic islet cells (11). Islets contain hundreds of different miRNAs, and great efforts have been made to identify those dysregulated in diabetes and to understand their function in these cells. This has been extensively reviewed by others and us during the past few years (12–14). Nevertheless, the molecular and cellular mechanisms leading to the regulation of miRNA expression and action in these cells remain elusive. In this review, we aim to discuss the different molecular mechanisms that control miRNA function and their contribution to the regulation of miRNA expression and action in β-cells and islets. Given the central role that glucose and fatty acids play in β-cell survival and function, we will pay special attention to those mechanisms involved in nutrient-mediated regulation of miRNAs.

## Canonical MiRNA Biogenesis and Mechanism of Action

**Figure 1:**
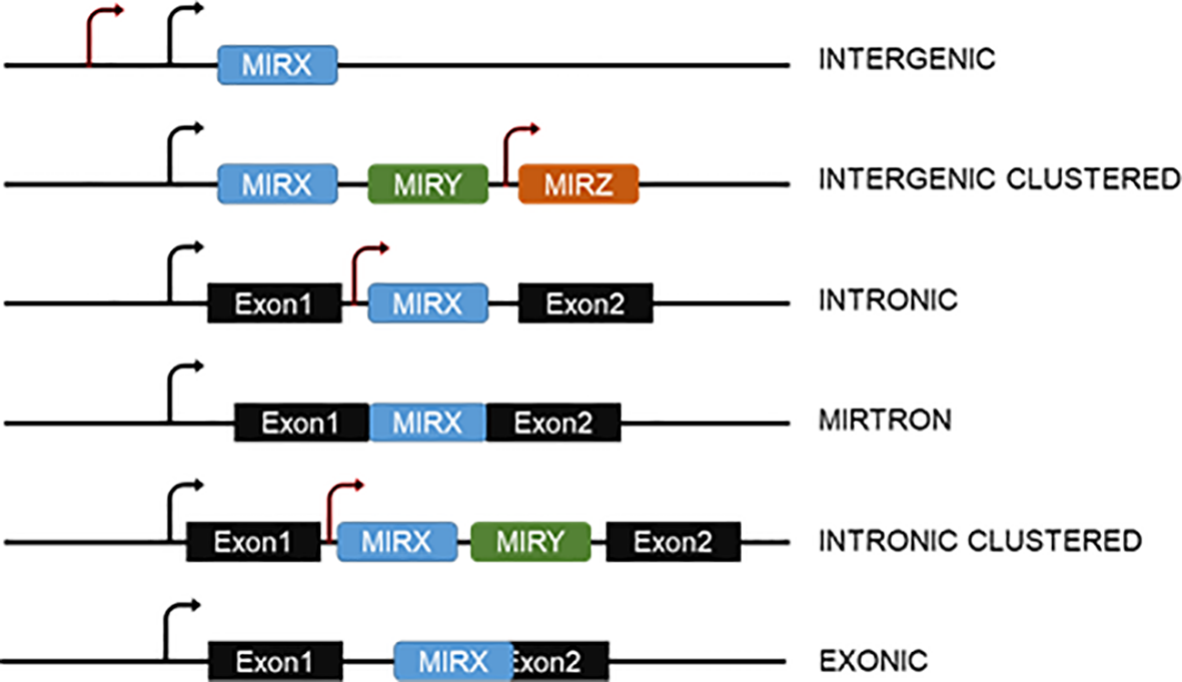
Genomic organization of miRNA genes. MiRNAs can be intergenic or be encoded within introns or overlapping exons of other genes. In both cases, they can be in clusters with other miRNAs. Mirtrons are generated from spliced-out introns and do not require the action of the Microprocessor. The arrows indicate the possibility of more than one transcription start site for both intergenic or intragenic miRNAs.

Genomic organization of miRNA genes occurs in multiple configurations (**Figure 1**). On one hand, animal miRNAs can be encoded by individual genes or in clusters with other miRNAs. While clustered, miRNAs are often transcribed as a common polycistronic transcript though miRNAs inside the cluster can additionally contain independent transcription start sites (TSS). On the other hand, individual and clustered miRNA genes can be intergenic or intragenic, as defined by their location within other protein coding genes, often within their introns. Adding further complexity, intragenic miRNAs can be expressed with and processed from the host gene or be generated independently from independent TSS. Alternative splicing of the host primary transcript can also lead to additional layers of regulation (15).

**Figure 2:**
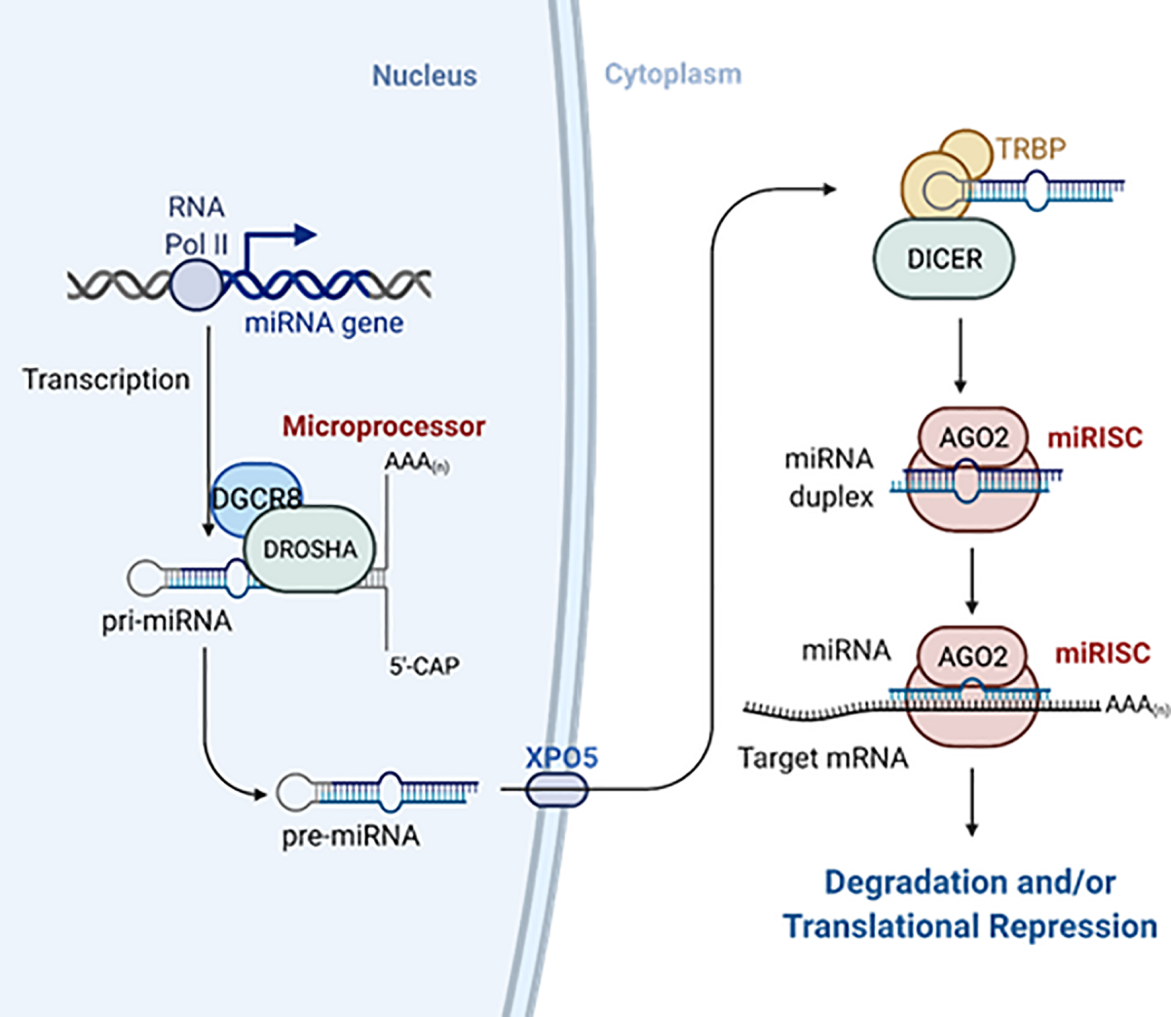
MiRNAs biogenesis. MiRNAs are transcribed as longer primary transcripts (pri-miRNAs) that are first processed in the nucleus by the Microprocessor complex to generate ~70-nucleotides pre-miRNAs that are exported to the cytosol by exportin-5 (XPO5). Pre-miRNAs are further processed by Dicer and TRBP into small RNA duplex. The mature miRNA is loaded with Argonaute proteins into the miRISC. Created with Biorender.com. See the main text for further details.

**Figure 3:**
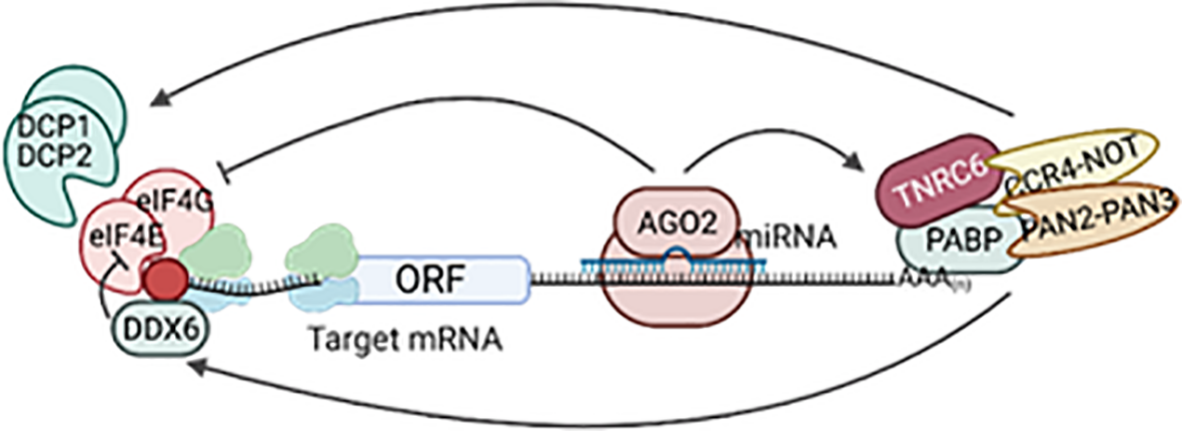
MiRNA mechanism of gene silencing. Following loading into miRISC, the miRNA guides the complex to partially complementary target mRNAs. MiRISC recruits TNRC6, PABPC, and deadenylase complexes CCR4-NOT and PAN2-PAN3, shortening the poly(A) tail. This can be followed by decapping and 5’-to-3’ exonucleolytic degradation and/or recruitment of the helicase DDX6 to inhibit translation. Other mechanisms of translational repression include inhibition of the recruitment of initiation factors (eIF4) and ribosome scanning. Created with Biorender.com.

Most mammalian miRNAs are transcribed by RNA polymerase II as capped and polyadenylated primary transcripts (pri-miRNAs) (16–18). Pri-miRNAs contain at least one hairpin with the mature miRNA sequence inside the stem and are often up to hundreds of kilobases long (16, 18, 19). Pri-miRNAs are recognized by the microprocessor complex, consisting of the RNAse III enzyme DROSHA and the double-stranded RNA binding protein DGCR8, between other proteins (20, 21). The microprocessor complex very rapidly cleaves the base of the hairpin to generate ~70 nucleotide miRNA precursors with a 2-nt 3’ overhang (pre-miRNAs) (22, 23). Pre-miRNAs are exported to the cytoplasm *via* exportin-5 (24, 25) where they are recognized and processed by the RNase III enzyme DICER into 20–25 nucleotides RNA duplex with ~2-nt 3’ overhang at each end, containing the mature miRNA (26, 27). The short miRNA duplex will bind a protein of the Argonaute (AGO) family in an ATP-dependent manner (28) that will promote the expulsion of one of the miRNA strands [the “passenger” or “star (*)”] (29). Which one of the strands is degraded depends on the orientation of the duplex within Argonaute as determined by the most 5’ nt sequence and the stability of the 5’ terminal pairing (30, 31). In some cases, both strands can work as mature miRNAs (32) (**Figure 2**). Consequently, DICER is dissociated from this complex known as the miRNA-induced silencing complex (miRISC). MiRNAs guide miRISC to partially complementary sequences in the 3’ untranslated region (3’UTR) of target mRNAs or, less frequently, their coding region (CDS) and, rarely, the 5’UTR (33). It is now well established that most miRNA binding sites contain extensive complementarity to nucleotides 2–8 of the miRNA (the “seed” region) and that an adenine opposite to position 1 of the miRNA enforces target recognition (34, 35). Nevertheless, non-canonical binding sites, with little or no seed-complementarity, can also mediate effective mRNA target recognition (33, 36, 37). MiRNA action results in silencing of gene expression through mainly two mechanisms in mammals: inhibition of translation and/or mRNA destabilization. MiRNAs recruit TNRC6 (orthologue to *Drosophila*’s GW182 and nematodes’ AIN-1/2), which interacts with the poly(A)-associated protein PABPC that recruits deadenylase complexes PAN2-PAN3 and CCR4-NOT resulting in poly(A) tail shortening, often followed by DCP1/DCP2-mediated decapping and 5’-to-3’ exonucleolysis by XRN1 of the target mRNA. TNRC6-CCR4-NOT can also recruit the helicase DDX6 resulting in inhibition of mRNA translation. Additional mechanisms contributing to inhibition of translation include the interference with initiation factor eIF4E and ribosome recruitment and scanning (**Figure 3**). The specific molecular mechanisms of repression and their relative contribution to gene silencing remain debated and can be cell- and miRNA-specific (9, 38). It is worth noting that while mRNA decay has been proposed to be the dominant mechanism for biologically meaningful gene silencing in mammalian cells (39), translational repression is the main mechanism regulating zebrafish development (40). Moreover, there are multiple examples of differentiated mammalian cells in which translational repression regulates the expression of key miRNA targets (41–45).

A given mRNA can be simultaneously targeted by several different miRNAs, and conversely, miRNAs usually target several mRNAs at the same time. This often results in small effects in the expression of several genes on the same biological pathway and contributes to fine-tune and reinforce cellular identity and states (46–48).

In addition, non-canonical mechanisms of miRNA biogenesis have been described that bypass one or more of the canonical steps mentioned above (49). For excellent comprehensive reviews on miRNA biogenesis and mechanisms of action, we refer the reader to Treiber et al. (50), Bartel (9), and Gebert and MacRae (38).

## Beta-Cell MicroRNAs and Their Regulation by Nutrients

Studies based on *Dicer* deletion at different stages during pancreas development and in the adult β-cell have extensively demonstrated that miRNAs are essential for endocrine cell differentiation (11, 51–53) and mature β-cell function and survival (54, 55). In the adult β-cell, miRNAs play a plethora of roles that range from control of insulin production (54, 56–59), insulin exocytosis (60, 61), apoptosis (62, 63), growth (64), repression of disallowed genes (55, 65, 66), and maintenance of β-cell identity (54, 67, 68). Others and we have comprehensively reviewed this work before (12, 13, 69), so here we will focus on the influence that nutrients have in islet miRNA expression and function and, more specifically, the molecular mechanisms underlying this regulation.

### Hyperglycaemia/Lipidaemia

Chronic hyperglycaemia impacts both β-cell function and survival (5) and is a critical contributor to the development of over diabetes (70). Hyperglycaemia exerts a strong effect on gene expression, which contributes to loss of β-cell identity and function in diabetes (71–73). Very recently, Ebrahimi et al. (74) performed RNAseq in islets from partially pancreatectomized rats subjected to different levels of hyperglycaemia and demonstrated that whereas changes in gene expression correlated with diabetes severity, even a very mild hyperglycaemia was sufficient to alter the expression of genes important for β-cell identity, insulin secretion, and mitochondrial function (74).

Obesity is strongly associated with insulin resistance and increased risk of T2D (75), and chronic exposure to free fatty acids (FFA), specially saturated fatty acids, leads to lipotoxicity that affects β-cell functionality and survival and contributes to the development of hyperglycaemia (76).

Whereas Goto-Kakizaki (GK) rats represent a non-obese model of T2D, db/db and ob/ob mice spontaneously develop severe hyperphagia leading to obesity and T2D (77). These models have been widely used to determine, both by high-throughput and targeted experiments, whether miRNAs contribute to glucolipotoxicity. Other studies have assessed the expression of miRNAs in islets of mice fed a high-fat diet or in cell lines and murine or human islets cultured under lipotoxic and/or glucotoxic conditions. **Table 1** contains a comprehensive list of glucose and fatty acid-regulated miRNAs and a summary of their key functions in pancreatic β-cells/islets. Nutrient-regulated miRNAs, such as for example miR-130a/d and miR-152 (81, 95), are also often dysregulated in diabetes in humans (**Table 1**, “Human T2D” column), supporting their contribution to the development of the disease. On the other hand, it remains contested whether the expression of other glucose-regulated miRNAs with important roles for β-cell function is dysregulated in T2D. For example, β-cell-specific miR-375 is an important regulator of β-cell mass and insulin secretion (153, 156, 168–170) upregulated by glucose in murine cell lines and islets, as well as in islets from ob/ob mice (64, 156). Nevertheless, only one study reported increased miR-375 in pancreatic tissue from T2D human donors (154) with most studies finding identical expression in islets from T2D and healthy human donors (95, 122, 123) and in other murine models of diabetes (81, 97). Another example is miR-184, an important mediator of compensatory β-cell secretion and expansion during insulin resistance in the face of obesity and pregnancy (64, 119). MiR-184 is strongly downregulated by glucose in MIN6 cells and mouse, rat and human islets (64, 97, 118), reduced in islets of prediabetic, db/db mice and mice fed a HFD (97) and strongly upregulated in islets of mice starved for 24 h or kept on a (low-sugar) ketogenic diet (64). Nevertheless, although Tattikota et al. observed downregulation of miR-184 in islets from human T2D donors, our group failed to confirm these differences, as so did others, perhaps concealed by the sex-dimorphism observed in miR-184 expression (118, 122, 123).

**Table 1:** Glucose/lipid regulated islet miRNAs.

Name	High glucose	Fatty acids	Diet	Animal model	Human T2DM	Function
***miR-107-3p***	↑ MIN6 (78)			↑ GK rat (79)	↑ In peripheral blood (80)	Unknown
***miR-124-3p***	↓ Rat islets (24 h) (81)↑ MIN6 (78, 81)			↑ GK rat (81)	↑ (82)	Negative regulation of GSIS (82)
***miR-125b-5p***	↑ Human islets ↑Mouse islets (83)		↓Ketogenic (Mouse islets) (83)		↑ In peripheral blood (84)	β-cells identity maintenance by repressing c-Maf (85) Promotion of β-cells survival by targeting Dact1 (86)
***miR-126-5p***				↑ B6 and BTBR *ob/ob* mouse (87)	↓ In peripheral blood (88–90) ↑ In peripheral blood (91, 92) ↓ (80)	Unknown
***miR-127-3p***	↓ MIN6 (24h) (93)		↓HFD (Mouse islets) (93)	↑ GK rat (81)	↑ In islets of glucose-intolerant individuals (94)	Negative regulation of β-cell proliferation and GSIS (93)
***miR-130a-3p***	↑ Human islets (95) ↑ GK rat islets (95) ↓ Wistar rat islets (81)		↓ HFD and HSD (Rat circulation) (96)	↑ GK rat (95)	↑ (95)	Negative regulation of GSIS and insulin content (95)
***miR-130b-3p***	↑ Human islets (95) ↑ GK rat islets (95)		↑ HFD (Mouse islets) (97)	↑ GK rat (95) ↑HFD-induced mouse model (97)	↑ (95)	Negative regulation of GSIS and insulin content (95)
***miR-132-3p***	↑ GK rat islets (24h) (81)	↑ Palmitate (Rat islets under glycolipotoxic conditions for 48 and 72h) (97)	↑ HFD (Mouse islets) (97)	↑ GK rat islet (81) ↑ B6 and BTBR *ob/ob* mouse (87) ↑ *db/db* mouse (97) ↑ HFD-induced mouse model (97)	↑ In peripheral blood and peripheral tissues (80)	Negative regulation of insulin secretion (98)
***miR-133a-3p***	↑ Human islets (99)			↑ B6 and BTBR *ob/ob* mouse (87) ↑ ZFD rat (100)	↑ In peripheral blood (101)	Negative regulation of insulin biosynthesis (99)
***miR-141-3p***			↓ HFD (Mouse islets) (102)			β-cell survival (102)
***miR-142-5p***	↓ Rat islets (24h) (81)			↑ GK rat (81)	↑ In peripheral blood (miR-142-3p) (80)	β-cell survival (103)
***miR-143-3p***				↑ *db/db* mouse pancreatic tissue (104)		Impairs glucose metabolism (104)
***miR-145-5p***				↑ *db/db* mouse pancreatic tissue (104)		Negative regulation of GSIS (105)
***miR-146a-5p***	↓ Human islets (99)	↑ Palmitate (Rat islets) (63) ↑ Palmitate (MIN6B1) (63)		↑ *db/db* mouse (63, 97) ↓ HFD, STZ-induced rat (islets and circulation) (106)	↑ In peripheral blood (107–109) ↓ In peripheral blood (110, 111)	β-cells survival (63, 97)
***miR-152-3p***	↑ Human islets (95) ↑ GK rat islets (95)			↑ GK rat (95) ↑ B6 and BTBR *ob/ob* mouse (87)	↑ (95) ↓ Peripheral blood (112)	Negative regulation of GSIS and insulin content (95)
***miR-153-3p***	↑ Mouse islets (72h) (113) ↑ MIN6 (4h)			↑ *db/db* mouse (114)		Negative regulation of GSIS and KCL stimulated insulin secretion (113, 115)
***miR-15a-5p***	↓ Mouse islets (1 h) ↑ Mouse islets (prolonged exposure) (116)				↓ In peripheral blood (88)	Positive regulation of insulin biosynthesis (116)
***miR-17-5p***	↓ Rat and mouse islets (at weaning) (117)		↓ HFD and HSD (Rat circulation) (96)		↓ In peripheral blood (81)	β-cell fate acquisition and maturation (117)
***miR-184-3p***	↓ Human islets (118) ↓Mouse islets (64, 118, 119) ↓ MIN6 cells (119)	Unchanged—Palmitate (MIN6 cells) (119) ↓ Palmitate (Rat islets under glycolipotoxic conditions for 48 and 72h) (97)	↑ Ketogenic diet (Mouse islets) (64, 119) ↑ Fasting (Mouse islets) (119) ↓ HFD (Mouse islets) (97)	↓ *db/db* and *ob/ob* mouse (64, 97) ↓HFD-induced mouse model (97) ↓B6 and BTBR *ob/ob* mouse (87)	↓ (64)	Expansion of β-cells to compensate for insulin resistance and insulin secretion regulation (97, 120)
***miR-185-5p***				↑ B6 and BTBR *ob/ob* mouse (87) ↓ STZ mice islets (121) ↓ ZDF rat (100)	↓ In peripheral blood (121) ↑ (80)	Possible positive regulator of GSIS and β-cell proliferation. Protects from apoptosis (121)
***miR-187-3p***					↑ (122, 123)	Negative regulation of GSIS (123)
***miR-199a-3p***	↑ Mouse islets (124) ↑ MIN6 (124)	↓ Palmitate (Rat islets under glycolipotoxic conditions for 72h) (97)		↑ *db/db* mouse (97) ↑ GK rat (81)	↑ Peripheral blood (125)	Induces β-cell apoptosis (97)
***miR-199a-5p***	↑ Mouse islets (124) ↑ MIN6 (124)			↑ *db/db* mouse (97) ↑ GK rat (81)	↑ Peripheral blood (125)	Negative regulation of GSIS and insulin content (97)
***miR-200a/b-3p***			↓ HFD (Mouse islets) (102)	↑ *db/db* mouse (12 weeks, very diabetic) (102)	↓ In peripheral blood (126)	β-cell survival (102)
***miR-203-5p***		↓ Palmitate (Rat islets under glycolipotoxic conditions for 48 and 72h) (97)	↓ HFD (Mouse islets) (97)	↓ *db/db* mouse (97) ↓ HFD-induced mouse model (97) ↓ ZDF rat (100)		β-cell proliferation (117)
***miR-204-5p***				↑ *ob/ob* and *db/db* mouse (127, 128)	Unchanged (122)	Insulin transcription regulation (127) Apoptosis and ER stress (129–131) Negative regulator of insulin exocytosis (130)
***miR-206-3p***			↑ HFD (Mouse islets) (132)		Unchanged (133)	Unclear
***miR-210-5p***			↓ HFD (Mouse islets) (97)	↓ *db/db* mouse (97) ↓HFD-induced mouse model (97) ↑ ZDF rat (100)		Unknown
***miR-212-5p***	↑ Rat islets (24 h) (81)			↑ GK rat (81) ↑ B6 and BTBR *ob/ob* mouse (87)		Regulator of GSIS and GLP-1 induced insulin secretion (134)
***miR-21-5p***				↑ *db/db* mouse (97)	↑ In peripheral blood (for miR-21) (91) ↓ In peripheral blood (for miR-21-5p) (92) ↑ In islets of glucose intolerant individuals (94)	β-cell proliferation and survival (135, 136)
***miR-223-3p***	↑ MIN6 (137)		↓ HFD (Mouse islets) (97)	↓ HFD-induced mouse (97)	↓ In peripheral blood (88, 138)	β-cell proliferation and GSIS (137)
***miR-25-3p***	↓ Rat and mouse islets (at weaning) (117)		↓ HFD and HSD (Rat circulation) (96)		↑ In peripheral blood of T1DM (139, 140)	Negative regulation of GSIS (58) Possibly regulate β-cell proliferation (141, 142)
***miR-26a-5p***			↓ HFD (Mouse islets and serum) (143)	↓ *db/db* mouse (97) ↓ HFD-induced mouse (islets and serum) (143)		Promotes β-cell differentiation (144) Positive regulator of insulin gene expression (54, 78) Positive regulator of insulin secretion under HFD (143)
***miR-296-5p***	↓ MIN6 (78)					Unknown
***miR-297-5p***		↓ Stearic acid (Mouse islets) (145) ↓ Stearic acid (Beta-TC6 cells) (145)				β-cell survival (145)
***miR-29a-5p***	↑ Human islets (146) ↑ Rat islets (146) ↑ INS-1E cells (146)			↓ HFD, STZ-induced rat (islets and circulation) (106)	↓ In peripheral blood (88)	Negative regulation of GSIS and β-cells proliferation (146)
***miR-29b-3p***	↑ Rat and mouse islets (at weaning) (117)		↓ HFD and HSD (Rat circulation) (96)		↓ In peripheral blood (138)	β-cell differentiation, maturation and identity maintenance (117) Insulin signalling (147)
***miR-30a-5p***	↑ Rat islets (3 days exposure to glucotoxicity) (148) ↑ MIN6 (78) ↑ INS-1E cells (148)		↓ HFD and HSD (Rat circulation) (96)	↑ *db/db* mouse (148)	↑ In peripheral blood (88)	Mediator of glucotoxicity, negative regulator of GSIS (148)
***miR-30d-5p***	↑ Mouse islets (149) ↑ MIN6 (78)			↓ *db/db* mouse (149) ↓ HFD, STZ-induced rat (islets and circulation) (106)	↑ In peripheral blood of glucose-intolerant individuals (106) ↓ In peripheral blood (106)	Regulation of insulin gene expression but not secretion (78) Favours insulin production by promoting Mafa expression (149)
***miR-335-5p***	↑ Rat islets (1h) ↓ Rat islets (24h) (81)			↑ GK rat (81)		Negative regulation of insulin exocytosis (150)
***miR-338-3p***			↓ HFD (Mouse islets) (97, 151)	↓ *db/db* mouse (151) ↓ HFD-induced mouse model (97)		Islet cell proliferation (151) Compensatory expansion of β-cell during insulin resistance (97)
***miR-345-5p***					↑ (123)	Unknown
***miR-34a-5p***		↑ Palmitate (Rat islets) (63) ↑ Palmitate (MIN6B1) (63) ↑Stearic acid (Mouse islets) (152) ↑ Stearic acid (INS-1) (152)	↑ HFD - both in stearic and palmitic acids (Mouse islets) (152)	↑ *db/db* mouse (63, 97) ↑ B6 and BTBR *ob/ob* mouse (87)	↑ In peripheral blood (84, 91, 108)	β-cells survival and proliferation (63, 97, 135)
***miR-34b-5p***				↑ B6 and BTBR *ob/ob* mouse (87)		Unknown
***miR-375-3p***	↓ Rat islets (2 h) (153) ↑ Rat islets (72 h) (153) ↓INS-1E (1–48 h) (153)		↑ HFD (Mouse islets) (64) ↑ HFD and HSD (Rat circulation) (96)	↓ GK rat (153) ↑ *ob/ob* mouse (64)	↑ (154, 155) Unchanged (81, 91, 95) (82) ↓ In peripheral blood (88)	Maintenance of β-cell mass (156) Glucose regulation of insulin gene expression (153)
***miR-376a-3p***	↓ Rat islets (24h) (81)			↑ GK rat (81)	↓ (122)	Apoptosis (122)
***miR-383-5p***	↓ MIN6 (157)	↓ Palmitate (Rat islets under glycolipotoxic conditions for 48 and 72 h) (97)	↓ HFD (Mouse islets) (97)	↓ *db/db* mouse (97) ↓HFD-induced mouse model (97)	↓ In serum (157)	Ameliorates hyperglycaemia-mediated apoptosis (157)
***miR-409-3p***	↑ Rat islets (24h) (81)			↑ GK rat (81)	↑ In peripheral blood (gestational diabetes mellitus, miR-409-5p) (158)	Regulator of apoptosis and GSIS (159)
***miR-429-3p***			↓ HFD (Mouse islets) (102)	↑ *db/db* (12 weeks, very diabetic) (102)		β-cell survival (102)
***miR-433-3p***	↓ MIN6 (24 h) (160)			↑ GK rat (81)		Negative regulation of β-cell growth (160)
***miR-451-5p***			↑ HFD (Mouse islets) (151)			Unknown
***miR-484-5p***	↓ MIN6 (78)					Unknown
***miR-690-5p***	↓ MIN6 (78)					Likely to impair maturation and insulin biogenesis (161)
***miR-708-5p***	↑ Rat islets ↓ Rat islets (24 h) (81)			↓ GK rat (81)		Negative regulator of GSIS and cell growth (162)
***miR-802-5p***			↑HFD (Mouse islets) (97)	↑ *db/db* mouse (97) (163) ↑HFD-induced mouse model (97)		Negative regulator of insulin expression (164) Impairs glucose metabolism` (163)
***miR-9-5p***	↑ Mouse islets (1 h after IP glucose injection) (165) ↓ Mouse islets (4 h after IP glucose injection) (165)				↑ Peripheral blood of pre-T2DM and T2DM (166) ↓ In peripheral blood (88)	Negative regulation of GSIS (60, 165, 167)

The table shows the direction of the glucose and fatty acid–dependent regulation by glucose and/or fatty acids (↑: upregulated, ↓: downregulated with high glucose and/or high fatty acid concentration, columns 2 and 3) in rodent (orange) and human (blue) cell lines (pale colour) and islets (dark colour). Column 4 (Diet) represents whether changes in miRNA expression have been observed in islets of rodents fed specific diet (high fat: HFD, high sugar: HSD, or ketogenic) and column 5 (Animal models) in murine models of diabetes. Column 6 indicates changes in expression associated with T2D in human islets or, where indicated, in the circulation. Column 7 briefly indicates the main proposed miRNA function in islets/β-cells.

### Low-Protein Diet

Feeding a low-protein (LP) diet during gestation represents a model of intrauterine growth restriction (IUGR) characterized by decreased placental leucine transport and reductions in essential amino acids in dams (171). Alejandro et al. (172)found that several miRNAs, including miR-7, -199a-3p, miR-152 and miR-342-5p were increased and others, such as miR-30b, miR-487b and miR-224, decreased in adult islets from mice born to LP-fed dams. Although the function of all these miRNAs was not interrogated, the authors demonstrated that miR-199a-3p and -342 regulated mTOR protein levels and insulin secretion in β-cells and their inhibition was able to rescue the secretory defects observed in these islets.

In another study, altered miR-375 levels were increased in neoformed islets from foetuses and in adult islets from the progeny of female dams fed a LP diet during pregnancy. These islets had impaired β-cell mass and function that was rescued by restoration of miR-375. Of note, an additional 43 miRNAs were upregulated while four were downregulated in the foetal pancreas of LP-fed mothers (173). In a similar study, Su et al. identified upregulation of miR-15 in offspring of LP-fed dams, and the inhibition of this miRNA corrected the defects in beta-cell proliferation observed in the islets (174). More recently, Alejandro and colleagues assessed the effect of the LP diet administered only during the last week of gestation. This was sufficient to induce glucose intolerance in offspring at age older than 12.5 weeks and to increase the sensitivity of HFD-mediated diabetes. On the contrary to what they had previously observed when LP diet was administered during the whole pregnancy, these defects occurred without changes in mTOR and resulted in dysregulation of a completely different subset of miRNAs, including miR-342, miR-143, miR-338, miR-335, and miR-184 (175). It is worth noting that even though very little is known on the effects of LP diet in maternal islet miRNAs, de Siqueira et al. observed a reduction in miR-124a that may contribute to defects in insulin release, possibly *via* regulation of the exocytotic machinery (176). Nevertheless, these studies were performed in murine models, and therefore further investigation is required to determine whether these effects are sustained in humans.

### Weaning

It has been demonstrated that the nutritional changes associated with weaning are essential to trigger complete β-cell maturation, develop β-cell capacity of compensatory proliferation, and improve glucose-stimulated insulin secretion (177). Interestingly, the expression of several miRNAs such as miR-25, miR-17, and miR-29b changes during this process and may contribute to the improvement in glucose-induced secretory response (117).

It is important to acknowledge that there is often little overlap in the differentially expressed miRNAs under diabetic/glucolipotoxic conditions between studies. Underlying these discrepancies are the use of low number of samples (specially for human islets), heterogeneity of the samples (i.e., origin, purity, sex, age, and other characteristics of the donors), experimental conditions (i.e., concentration and duration of the glucolipotoxic insult, or diet composition), or the use of different detections systems (i.e., qPCR, arrays, high-throughput sequencing). Moreover, β-cell lines represent a de-differentiated system that only partially preserves the original characteristics of primary β-cells. Additionally, cell lines are removed from the natural tissue architecture and therefore lack cell-cell interactions with other endocrine, and non-endocrine, cells. Many studies have on the other hand been performed in full islets, where β-cells only represent a proportion (50–80%, humans-mice) of the cellular population. Islets might also respond differently to treatments in isolation or to diets administration *in vivo*. Finally, even though mature miRNA sequences are generally highly conserved amongst mammals, different levels of expression often occur between homologous tissues of mice and humans, especially for late-emerging miRNAs (178). Rodent and primate islets not only differ in innervation and architecture, but the β-cells contain significant transcriptome differences that involve species-specific transcription factors and long non-coding RNAs (lncRNAs) (179). It is therefore imperative that nutrient-dependent regulation of miRNA expression is validated in human islets, facilitated by the significant improvement of human islets availability during the past decade. Moreover, “humanized” mouse models for the study of human β-cell function *in vivo* are now well established in several laboratories around the globe. In these models, human islets are transplanted into mice, often following drug-mediated elimination of their own islets. This approach has been previously used to identify human islet miRNAs associated with β-cell destruction in mouse models of T1D. The effect of experimental diets fed to the recipient mice could also be investigated in the transplanted human islets. Whereas standard protocols involve transplantation under the renal capsule and the effects on transplanted-islet miRNAs can only be measured at specific end-points, recent developments use transplantation in the anterior chamber of the eye, which allow *in vivo*, longitudinal imaging of the implanted islets, often exploited to interrogate functionality and engraftment (180). Recent advances in miRNA monitoring *in vivo*, such as the use of fluorescently labelled miRNA activity reporters or molecular beacons which activity depends on the presence of the miRNA (181), could be incorporated to allow the interrogation of miRNA function in the transplanted beta cells.

## Molecular Mechanisms Underlying Nutrient-Mediated Regulation of miRNA Action

**Figure 4:**
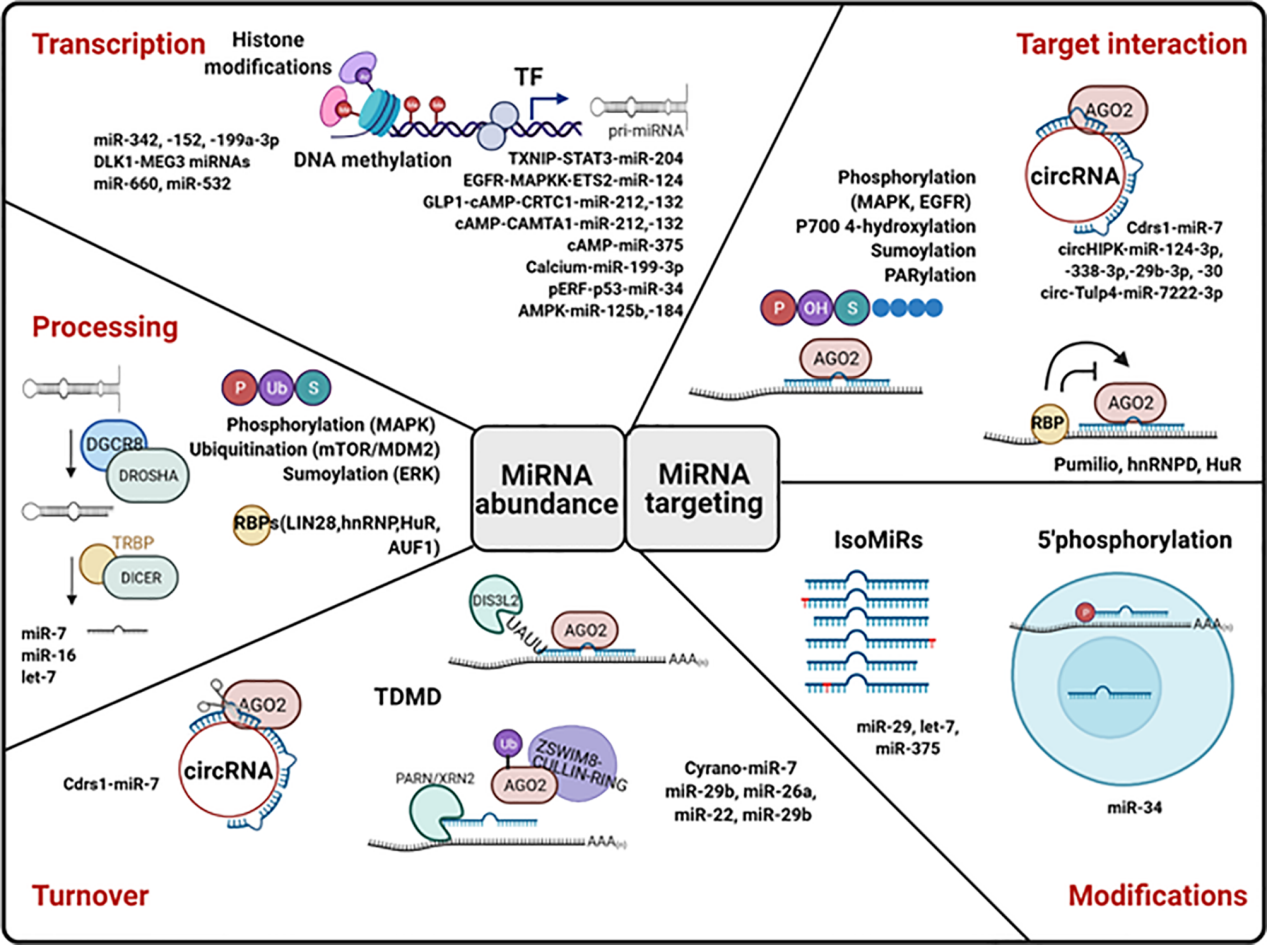
Molecular mechanisms of miRNA regulation. MiRNA levels can be control at the level of transcription, processing, and stability. MiRNA transcription is regulated by epigenetic modifications (histone modifications and DNA methylation) and by the action of transcription factors (TF). The action of proteins important for RNA processing is regulated by post-translational modifications (P, phosphorylation; U, Ubiquitination; S, Sumoylation) and by RNA-binding proteins (RBPs). Circular RNAs (cirRNAs) can promote miRNA degradation. TDMD, target-directed miRNA degradation, can occur dependently or independently of miRNA tailing and trimming. The capacity of miRNAs to bind and repress their targets can be modulated by miRNA modifications and by RBPs, lncRNAs, and circRNAs. Examples of islet miRNAs regulated at each level are included in the figure. Created with Biorender.com. See text for more details.

Despite the great effort dedicated to identify glucose- and diabetes-regulated miRNAs, little information is available regarding the cellular and molecular mechanisms underlying changes in islet miRNA expression. Cellular expression of mature miRNA is determined by the rates of miRNA transcription, processing, and decay, each of which can be globally or specifically regulated. Moreover, miRNAs’ capacity to bind and silence their target can be modulated by additional factors such as post-transcriptional modifications and interaction with proteins and other non-coding RNAs (**Figure 4**).

### MiRNAs Transcription

As is the case for messenger RNAs, the transcription of each pri-miRNA is tightly controlled by epigenetic and transcription factors. Epigenetic regulation of miRNA expression has been widely studied in tumours and involves DNA methylation and histone modifications that often contribute to miRNA dysregulation during tumour progression (182). It is well established that miRNAs form tightly coordinated regulatory networks with transcription factors, which contribute to reinforce cellular states (47).

Several examples of nutrient-dependent regulation of miRNA transcription have been described in pancreatic islets. MiR-204 is an intronic miRNA located within and sharing a common promoter with *Trpm3* (183) and upregulated in islets of ob/ob (127) and db/db mice (128). In pancreatic islets, TXNIP stimulates the expression of both miR-204 and TRMP3 through, at least in part, inhibition of STAT3 (signal transduction and activator of transcription 3) activity (127). MiR-204 blocks insulin production *via* direct targeting of *Mafa*, contributing to the development of diabetes (127), and its inhibition improves glycaemic control in db/db mice (128). Another interesting example is miR-124 (miR-124-3p/miR-124a), which is a negative regulator of insulin secretion (82) upregulated by glucose in MIN6 cells (78), islets of GK rats (81), and T2D human islets (82). In a recent study, Yang and colleagues demonstrated that β-cell miR-124a expression is negatively regulated by EGFR signalling through MEK/ERK and PI3K/AKT downstream signalling cascades leading to ETS2 (E26 transformation-specific 2) activation and association with miR-124a promoters on chromosomes 2,3 and 14 (184). Glucose (185), HFD, and pregnancy (186) have been previously shown to modulate EGFR signalling, and it is therefore tempting to hypothesize that this EGFR-MEK/PI3K-ETS2 pathway contributes to the regulation of miR-124 by nutrients. Interestingly, miR-124 targets ionotropic glutamate receptors iGluR2 and iGluR3 in islet α-cells, and the expression of this miRNA and iGluR2/3 was strongly inhibited and upregulated, respectively, following fasting. Thus, miR-124 might additionally contribute to glucotoxicity by impairing glucagon release by α-cells (187).

Recently, Werneck-de-Castro et al. explored the mechanisms underlying the regulation of miR-199-5p and miR-199-3p in murine β-cells. As previously mentioned and summarized in **Table 1**, these miRNAs are upregulated in islets from mouse models of diabetes and in the progeny of LP-fed dams and have been implicated in impaired β-cell function and survival (81, 97, 172). Werneck-de-Castro et al. found that pri-miR-199 abundance is also regulated by glucose in MIN6 cells and mouse islets and that membrane depolarization-induced calcium release modulates *Mir199a-2* promoter activity and mature miR-199 expression acutely, though the exact molecular mechanisms remain to be elucidated (124).

Additionally, miR-34a is increased in INS-1 cells cultured with palmitate and stearic acid, as well as in islets from mice fed a high-stearic acid or a high-palmitic acid diet and db/db mice. Inhibition of this miRNA reduced lipotoxicity-induced islet β-cell death and rescued insulin content (152). These authors also demonstrated that pERK-p53 mediated miR-34a upregulation. They suggested that this occurs at the level of miR-34a transcription since p53 is a well-established transcriptional activator of this miRNA in cancer and other cell types (188, 189).

Other interesting examples of transcriptional regulation are miR-132 and miR-212. Both miRNAs are upregulated in GK rats (81) and induced by glucagon-like peptide 1 (GLP-1) in mouse and human islets (134). GLP-1 is an incretin hormone produced by the L-cells in the gastrointestinal track upon oral nutrient administration and a potent insulin secretagogue (190). GLP-1 activates GLP-1R triggering the activation of adenylate cyclases and the generation of cAMP that promotes granules exocytosis (191). Malm and colleagues demonstrated that the CREB (cAMP-responsive element binding protein) transcriptional co-activator CRTC1 (cAMP-regulated transcriptional Co-activator-1) and the CRTC inhibitors SIK1-3 (salt-inducible kinases) mediate cAMP-co-regulation of miR-212/miR-132 in rat β-cells (192). In a separate study (193), the same group showed that hyperglycaemia increased the expression of both mature and primary miR-212/-132. Interestingly, knockdown of calmodulin binding transcription activator 1 (*Camta1*) increased the expression of primary and mature miR-212/132 in rat islets and clonal β-cell and reduced the increase in pri-miR-212/132 levels mediated by the adenylate cyclase activator foskolin (193). Intriguingly, pri-miR-212/132 remained unchanged following stimulation with GLP-1, perhaps due to the complex kinetics of cAMP signalling in these cells (194). To the best of our knowledge a high-throughput screen of miRNAs regulated by GLP-1 (and/or cAMP) in islets has not been performed. Nevertheless, mTOR upregulation and concomitant decrease of miR-7, miR-375, and miR-9 was also observed in islets of HFD-fed mice treated with the GLP-1 analogue exendin-4 (195, 196). Additionally, Keller et al. found that miR-375 promoter occupancy by Pol II was reduced with cAMP agonists and concluded that cAMP represses miR-375 at the transcriptional level. Of note, this regulation occurred *via* PKA and was not modulated by glucose. Interestingly, miR-375 expression is downregulated in islets from GK rats, in INS1 cells cultured at high-glucose concentrations during 1 h–4 days and in rat islets cultured during a short period of time (2 h) at high-glucose concentration (81, 153). Nevertheless, rat islet miR-375 was upregulated upon 72 h of exposure to high glucose (153). Similar differences were observed for other miRNAs involved in the control of insulin secretion and content such as miR-132, miR-335, and miR-15 (81, 116) and might reflect the dual role of short- and long-term glucose in pancreatic β-cell function and survival. In an earlier study, Keller et al. found that β-cell specific transcription factors, namely, *Pdx1* and *NeuroD1*, bind the proximal promoter and distal enhancer of *MIR375*. Glucose stimulates Pdx1 activation by the PI-3 kinase pathway (197), whereas miR-375 represses PI 3-kinase signalling to limit glucose stimulation of insulin gene expression (153), resulting in a negative-feedback loop that may contribute to the effects of glucose in miR-375, Pdx-1, and insulin expression.

Finally, our group showed that islets with β-cell selective deletion of AMP-activated protein kinase (βAMPKdKO) contain decreased and increased levels of both mature and primary miR-184 (118) and miR-125b miRNAs (83), respectively, suggesting regulation at the transcriptional level. Nevertheless, further studies are needed to fully elucidate the transcription factors involved in this regulation and to interrogate whether additional mechanisms are also involved. Several transcription start sites (TSS) in two different *loci* have been annotated in other cell types for miR-125, while miR-184 TSS remains to be fully annotated, which complicates these studies.

Of note, the majority of these studies have been performed in rodent islets/β-cells, so it is yet to be determined whether the transcription of these miRNAs is regulated in a similar manner in humans.

DNA methylation and histone modifications have been extensively demonstrated to control islet gene expression and secretory function (198), and both are altered during lipoglucotoxicity in β-cells (199, 200) and in T2D [reviewed in (201)]. Nevertheless, there is limited knowledge on their contribution to regulation of islet miRNA expression. Alejandro et al. (172) noted that the majority of miRNAs upregulated in islets from mice born to LP-fed mice are regulated by methylation of their promoters (miR-342, -152, and -199a-3p-a1) and that miR-199a-3p-a2 and -7a contain E-boxes, so could potentially be transcriptionally targeted by Ngn3 and NeuroD/β2. Kameswaran et al. (122) detected a cluster containing 54 miRNAs (DLK1-MEG3) highly expressed in β-cells and strongly downregulated in islets from T2D human donors. This cluster is maternally imprinted, and these authors observed that DNA methylation in a differentially methylated region responsible for the maternal imprinting was increasingly methylated in the T2D islets. Differential methylation between male and female human pancreatic islets was also associated with the levels of miR-660 and miR-532 (202). More studies are required to elucidate the contribution of these mechanisms to nutrient-mediated regulation of islet miRNA expression.

#### The Challenge of Studying miRNA Transcription

Although transcriptional regulation of a few specific islet miRNAs has been studied to some extent, as exemplified above, nutrient-mediated effect on β-cell miRNA transcription remains to be investigated in a high-throughput manner.

During the past few years, several methods have been developed to measure transcriptional rates genome-wide, many of which rely in enrichment in nascent mRNAs. Some examples are Nascent Elongating Transcript Sequencing (NET-seq), which uses immunoprecipitation to capture RNAs associated with PolII (203), and run-on-based assays such as Coordinated Precision Run-On and sequencing (CoPRO) (204), that use radio-labelled NTPs to tag nascent RNAs, which can be identified by high-throughput sequencing and provide active site location of PolII and cap status from single nascent transcripts.

Nevertheless, the use of these approaches to study miRNA transcription is subject to the availability of accurate pri-miRNA annotations. MiRNA transcription start sites (TSS) are often tissue-specific and conventional techniques for miRNA TSS identification such as 5’RACE, and RNAseq has been somewhat restricted by the quick processing of pri-miRNAs into mature miRNAs and by the fact that miRNA TSS can be located thousands of nucleotides away from the mature miRNA sequence. The quick improvement in the resolution of high-throughput sequencing technologies and the computational analysis (including deep machine learning) has more recently allowed the integration of epigenetic marks (H3K4me3, H3K9/14Ac, PolII) and cap analysis of gene expression (CAGE-seq) to more precisely map miRNA transcription start sites in a cell-specific manner (205–207). More recently, Liu et al. (206) used global nuclear run-on sequencing [GRO-seq (208)] and precision nuclear run-on sequencing (PRO-seq [209)] that provide sharp peaks around transcription start sites and continuous signal over active transcription regions to map the TSS of 480 intergenic miRNAs in 27 different human cell lines. Moreover, they integrated this information with ChIP-seq data from matched ENCODE tissues and generated a transcriptional circuitry that further demonstrated the complex interplay between TF and miRNA. Importantly, these authors generated a computational tool, mirSTP (http://bioinfo.vanderbilt.edu/mirSTP/), which can be utilized to map miRNA TSS in any cell type with available GRO/PRO-seq data. Although big consortium studies such as ENCODE do not normally generate data on pancreatic islets, which comprise a small proportion of the pancreas, the increased accessibility to protocols and systems for genome-wide profiling of epigenetic marks is allowing biologists worldwide to rapidly generate and share islet-specific data. These data will hopefully be used in the near future to precisely map β-cell/islet miRNA TSS and to shed more light into the transcriptional mechanisms by which nutrients control pri-miRNA expression in these cells. Additionally, imaging methods that use *in vivo* labelling of the sites of nascent transcripts [reviewed in (210)] can also be adapted for their use to study transcription of specific miRNAs in β-cells in response to nutrients *in vivo*.

It has been long established that miRNAs act cooperatively with other miRNAs and transcription factors that are themselves targeted by miRNAs, typically acting as hubs in regulatory networks. Over the past decade, fed by the increased availability of expression data and high-throughput miRNA target determination, important efforts have been made to develop *in silico* approaches that integrate this information to identify key regulatory networks (211). Nevertheless, most available networks are built based on gene-wide miRNA targeting and gene expression correlations, with very little input on regulation of miRNA gene expression. For example, miRNet (212) is a web-accessible tool originally generated to illustrate miRNA target interactions in which a given miRNA can target several genes and a gene can be targeted by several miRNAs. More recently, MiRNet 2.0 attempts to integrate transcription factor–miRNA interactions obtained from TransmiR (213) that incorporates publication-curated miRNA-TF as well as ChIP-seq identified interactions. MiRNet has also implemented a tissue-specific filter that restricts the cellular context based on miRNA expression profile, but profiles in pancreatic islets and/or beta cells have not been included. Very recently, Wong et al. (214) used miRNA profiling data of insulin-negative human tissue samples and (insulin-expressing) pancreatic islets and applied an unbiased, machine learning discovery approach to identify miRNAs associated with insulin mRNA that regulated insulin transcription. A similar networking method could be applied in the future to identify transcriptional regulators of the expression of specific islets miRNAs by integrating tissue-specific mRNA expression data.

### MiRNA Processing

As mentioned above, the abundance of mature miRNAs can strongly depend on their processing from pri- and pre-miRNAs. Both *Drosha* and *Dicer* were downregulated at the mRNA level in hyperglycaemic rat islets, though the impact on miRNA expression remained to be determined (74). On the contrary, proteomic analysis of isolated human islets cultured at high glucose concentration identified increased levels of DICER (215). On the other hand, peripheral blood mononuclear cells (PBMCs) from mice treated with metformin or caloric restriction contained higher levels of *Dicer1* mRNA due to increased binding of the RNA Binding Protein (RBP) AU-rich element-binding factor 1 (AUF1) (216). Of note, no changes in *Drosha* or *Dicer* expression were observed in βAMPKdKO islets (217), suggesting a glucose effect independent of AMPK and the need of further studies to clarify whether glucose regulate the miRNA processing machinery.

Both Microprocessor proteins and Dicer can suffer post-transcriptional modifications that modulate their activity. For example, DGCR8, DROSHA, and TRBP can be phosphorylated by mitogen-activated protein kinases (MAPKs), DROSHA can be ubiquitinated *via* mTOR/MDM2 activation, and DGCR8 sumoylation and stabilization can be stimulated by ERK [reviewed in (218)]. MAPK pathways play central roles in β-cells response to stress, including hyperglycaemia and lipotoxicity. Whether this results in post-translational modifications of the miRNA biogenesis machinery, though likely, remains to be investigated.

RNA binding proteins (RBPs) can bind to precursor miRNAs to alter their processing by the Microprocessor and Dicer, providing a sequence-specific level of regulation [see (219] for a comprehensive review). Some examples include the well-characterized protein LIN28, which can act both at the level of DROSHA in the nucleus or by impairing DICER-mediated processing in the cytosol (220, 221) and hnRNP A1 that can enhance pri-miRNA processing (222) but has a dual effect on pre-miRNA maturation (223, 224). Unfortunately, it is not currently known whether processing of specific miRNAs contributes to nutrients’ action in islets. As a proof-of-concept in HeLa cells, oleic acid induced miR-7 processing through remodelling of pri-miR-7 and inhibition of RBPs HuR and MSI2 binding and reduced miR-16 processing by an unknown mechanism (225). Also, it has been recently shown that Lin28a protects against β-cell destruction induced by streptozotocin and may enhance insulin secretion in a whole-body model of overexpression. Interestingly, MIN6 cells overexpressing this protein contained reduced levels of let-7 family members, though other miRNAs were not investigated (226).

### MiRNA Turnover

MiRNA half-life differs considerably between miRNAs, cell types, and importantly, cellular context, ranging from minutes to weeks (227–229). For example, neuronal miRNAs present faster turnover rates in comparison with other tissues, which reflects how crucial dynamic miRNA regulation is for the nervous system (183). It has been proposed that the base composition of the miRNA can itself impact degradation rate (183) and that miRNA stability can vary according to their binding to specific members of the Ago family (228). Nevertheless, the mechanisms responsible for miRNA turnover remain poorly understood. Addition or removal of nucleotides in the 3’ end of miRNAs (see *MiRNA Modifications* below), a process known as tailing and trimming, alters the stability of some, but not all, miRNAs (230). MiRNA degradation can be triggered by extensive (albeit incomplete) complementarity to target mRNA in a process known as target-directed miRNA-degradation (TDMD) (231). TDMD often involves 3’ terminal tailing and trimming of the miRNA, and it is a relevant mechanism contributing to neuronal miRNA regulation (232). Nevertheless, recent work by Mendell and Bartel labs have uncovered a tailing- and trimming-independent TDMD mechanism mediated by the ZSWIM8 Cullin-RING E3 ubiquitin ligase, which occurs in multiple cell types (233, 234). ZSWIM8-CRL binds to and targets for degradation AGO proteins exposing miRNAs for degradation by cytosolic RNAses, such as PARN and XRN2. XRN2 has already been demonstrated to mediate miRNA degradation in *C elengans* (235), although its role in mammalian cells might be more complex and leads to both miRNA degradation but also stabilization (236). On the other hand, the Perlman syndrome exonuclease DIS3L2 has been implicated in the degradation of oligouridylated miRNAs (237). Many of these proteins are components of the no-go and non-sense-mediated RNA decay pathway that were upregulated by pro-inflammatory cytokines in INS-1 cells to affect stability of Ins1/2 and other mRNAs, though miRNA expression was not interrogated (238).

Long non-coding RNAs can induce TDMD of miRNAs. This has been observed for miR-29b in Zebrafish (239) and for miR-7 in brain in zebrafish and mice (240). Interestingly, neuronal miR-7 was regulated not only by the lncRNA Cyrano but also, to a lesser extent, by Cdr1as, a circular RNA. In this model, miR-7 repressed Cdr1as *via* binding to multiple regions, forming a post-transcriptionally regulatory network important for neuronal function (240). Cyrano is poorly expressed in islets, and its deletion does not affect miR-7 expression (240). Nevertheless Cdrs1 has been shown to regulate insulin secretion and transcription in MIN6 cells and islets, allegedly by acting as a miR-7 sponge de-repressing its targets (59). Interestingly, *Cdrs1* is downregulated in islet from db/db and ob/ob mice (241).

To the best of our knowledge, the effect of nutrients on islet miRNA stability hasn’t been investigated. Stefan-Lifshitz et al. found that human islets exposed to cytokines showed changes in DNA methylation patterns and gene expression, including that of the exoribonuclease PNPase old-35 (PNTP1) that caused degradation of miR-26a and, to a lesser extent, miR-22 and miR-29b (242). This in turn increased 5-hydorxymethylcytosine levels *via* upregulation of ten-eleven-translocation 2 (TET2). It would be interesting to know whether this mechanism contributes to reduced miR-26a in db/db and HFD-fed mice (143).

As mentioned above, miR-204 and its host gene *Trmp3* are transcriptionally repressed by TNXIP/STAT3 in islets. Interestingly, miR-204 and *Trpm3* are also co-regulated in retina where not only rapid transcription but also fast decay allow for miR-204 rapid turnover during light-dark adaptation (183). Many of the studies on miRNA stability have focused on neuronal miRNAs, and it has been indeed suggested that neuronal mechanisms regulating miRNA degradation are unique compared to other tissues. Given that neurons and β-cells share multiple physiological and organizational characteristics as well as gene expression patterns (243), this represents a promising future area of study for islet biologists.

Some of the molecular approaches to estimate RNA synthesis mentioned above have been further engineered to measure RNA degradation rates. An example is transient transcriptome sequencing (TT-seq), which is based on sequencing following the short exposure of cells to the nucleoside analogue 4-thiouridine (4sU-seq) that is incorporated during transcription and allows isolation of labelled RNAs (244). The Ameres lab developed an optimized TT-seq protocol called Time-resolved RNA sequencing (SLAM-seq) to determine pri-miRNA transcription rates that, importantly, they combined with the rates of mature miRNA biogenesis (assessed by SLAM-small-seq) to determine miRNA processing in a high-throughput manner in a *Drosophila* cell line (228). Even though these approaches are normally limited to their use in cultured cells, 4-thiouracil can be administered by intraperitoneal or subcutaneous injection (245), opening the doors to its application to the study of miRNA kinetics in β-cells/islets *in vivo*.

### MiRNA Modifications

Any changes affecting miRNA sequence will have an impact on their capacity to recognize their targets, especially if those changes rely within the seed region. IsomiRs were first described by Morin et al. (246) and are microRNA isoforms originated from the same gene that differ in length and/or sequence. IsomiRs are usually generated by alternative Drosha/Dicer cleavage (247) but also by non-templated nucleotide addition (NTA) or RNA editing, and the proportion of IsomiRs expressed is cell- and tissue-specific (38, 248, 249). Alterations in the 5’ end of the miRNA tend to result in changes in their targeting properties, including potency and identity of the targets (248, 250). On the other hand, 3’ isomiRs usually present altered guide/passenger strand selection and stability and, to a lesser extent, targeting properties (247, 251). MiRNA precursors can be subject of deamination by ADAR1/2 that converts adenosine to inosine (249). Inosine shows guanosine characteristics, and thus A-to-I editing can alter miRNA biogenesis and stability, as well as target recognition (252, 253). Although unfrequently observed so far, A-to-I editing of miRNAs has already been shown to be biologically relevant for tumour progression (254, 255). The 3’ ends of mature miRNAs can also be subjected to adenylation or uridylation (256). Although not much data are currently available, the mechanism regulating adenylation involves, at least in some cases (i.e., liver miR-122, or miR-21 in tumours), the poly(A) RNA polymerases GLD2 and/or PAPD5 and the ribonuclease PARN (257, 258). Uridylation of miRNAs by TUT4 and TUT7 can both limit and expand target repression (259, 260) and can reposition and shift Dicer processing when occurring in the pre-miRNA (261).

**Figure 5:**
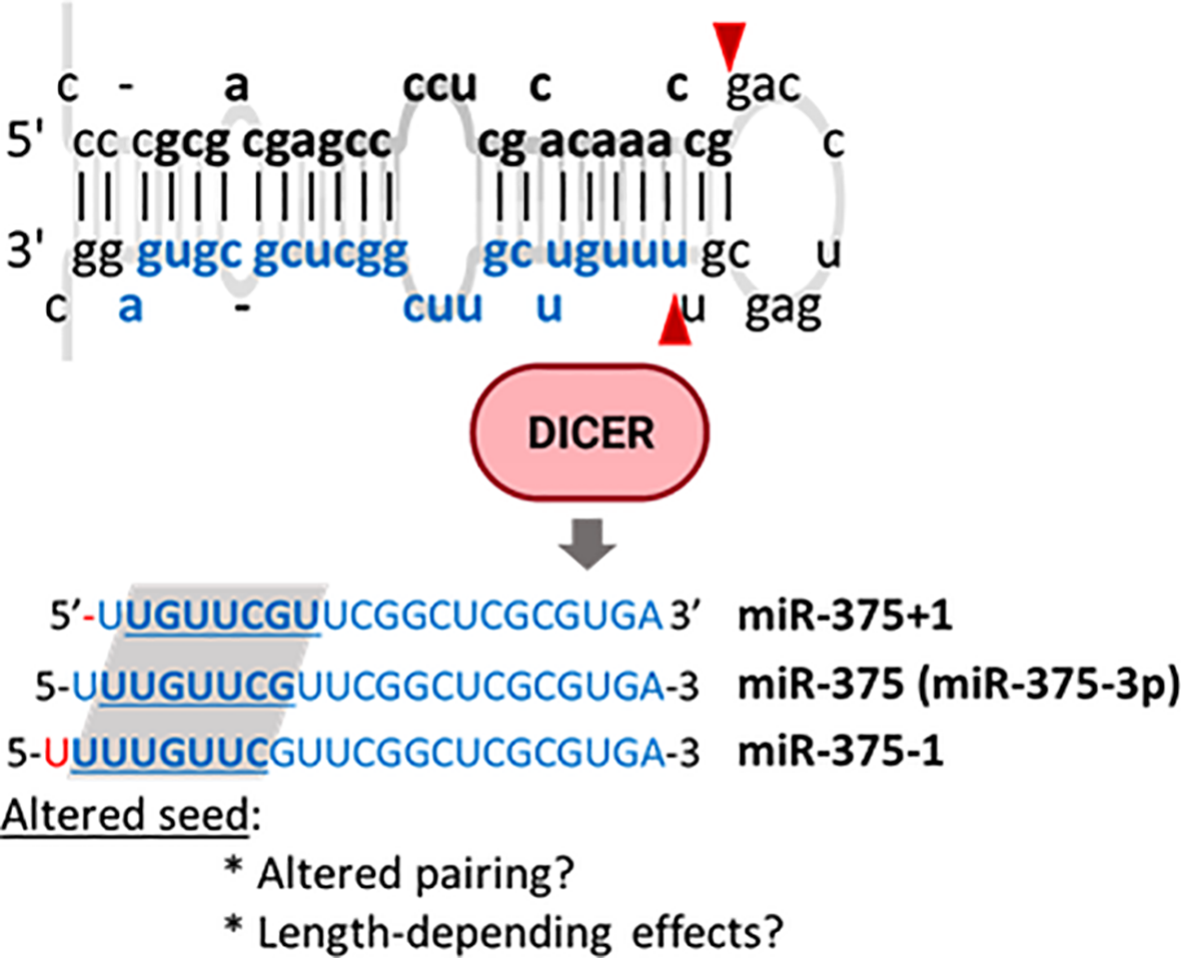
MiR-375 isomiRs identified in pancreatic islets. Pre-miR-375 may be alternatively processed by Dicer to generate isoMiRs with one additional (miR-375-1) or one less (miR-375+1) nucleotide in the miRNA 5’. These modifications have the potential to alter target recognition.

Baran-Gale et al. undertook a computational effort to profile isomiRs in small RNA sequencing data from MIN6 cells and human β-cells and islets and identified multiple 5’isomiRs. Some of the isomiRs with shifted 5’ positions (5’-shifted isomiRs), such as miR-375+1 and miR-375-1, were as abundant as the reference miRNAs (**Figure 5**). Further analysis suggested that some of these isomiRs, including miR-29, let-7, and miR-375, were predicted to target multiple alternative genes dysregulated in T2D. Experimental validation of the physiological relevance of these isomiRs remains to be provided, as well as further insights into the mechanisms leading to the generation of these and other miRNAs. HFD feeding, fasting, and glucose regulate the expression of ADAR2 by MAPK signalling in mouse islets and β-cell lines. ADAR2 is essential for adequate exocytosis, but its contribution to islet miRNA editing is unknown (262–264).

The 5’ miRNA phosphorylation/de-phosphorylation has emerged as an additional mechanism contributing to regulation of miRNA, though, to the best of our knowledge, it has so far only been observed for miR-34. In the absence of DNA damage, a pool of miR-34 lacking 5’phosphate remains inactive and not loaded into Ago. Nevertheless, ionizing radiation activates miR-34 by phosphorylation dependent of ATM (ataxia telangiectasia) and the RNA 5’kinase Clp1 (cleavage factor polyribonucleotide kinase subunit 1) (265). Of note, both ATM and miR-34 regulate β-cell damage response and survival (152, 266, 267).

### Regulation of miRNA-Target Accessibility

Target accessibility is a critical factor in microRNA function and can be regulated at different levels, including the interaction with proteins, non-coding RNAs, and subcellular localization.

#### Regulation of Protein Activity in miRISC

Ago proteins consist in four domains: the amino (N)-terminal domain, the PAZ (Piwi-Argonaute-Zwille) domain, the MID (middle) domain, and the PIWI (P-element induced wimpy testes) domain, as well as two interconnecting, linker domains (L1 and L2). The 5’ end of the miRNA is hold by the MID and PIWI domains, whereas the PAZ domain binds the 3’ of the miRNA (268). There are four Ago proteins (1–4) present in mammals, of which Ago2 is the most abundant and the only one retaining catalytic activity (269). Ago proteins can undergo multiple types of post-translational modifications. Phosphorylation of the L2 linker or the MID and PIWI domains have been widely observed and modulate the activity of the protein by altering their miRISC assembly capabilities, stability, and/or trafficking to exosomes [reviewed in (38)]. Other post-translational modifications include ubiquitination and Lys402 sumoylation to destabilize Ago2 (270, 271), Pro700 hydroxylation to increase Ago2 stability (272), and PARylation [poly(ADP-ribosylation)] that reduces target accessibility (273, 274).

Tattikota et al. demonstrated that reduction of AGO2 during pregnancy and obesity-mediated β-cell compensation is required for compensatory proliferation. They also showed that loss of Ago2 in an ob/ob background results in the upregulation of mRNA targets of the abundant islet miRNA miR-375 (64). As previously mentioned, these authors found that Ago2 downregulation occurred as a consequence of direct targeting by the glucose-regulated miRNA miR-184. Other mechanisms contributing to glucose-mediated regulation of Ago2 content or post-transcriptional modifications have not been investigated in pancreatic β-cells.

#### RNA Binding Proteins (RBP) Can Modulate miRNA Targeting

Following the demonstration by Kedde et al. in 2007 that dead end 1 (Dnd1) counteracts the function of several miRNAs in human and zebrafish cells by binding to U-rich regions in the mRNAs and impeding miRNA accessibility (275), many other RBPs have been demonstrated to play essential roles in controlling miRNA-target binding. Pumilio represents another example of an RBP that limits miRNA accessibility to the target mRNAs (276), whereas HNRNPD (AUF1) enhances miRISC interactions in HeLa (277) and HuR can both antagonize or promote miRNA function (278, 279). HuD is a member of the Hu family highly expressed in β-cells regulated by glucose and insulin receptor signalling. In β-cells, HuD binds to the *Ins* 5’UTR and promotes its translational repression (280). On the contrary, HuD regulates β-cell death and mitochondrial function by binding to the 3’UTRs and increasing the translation of ATG5 (autophagy-related gene 5) and Mfn2 (Mitofusin 2), respectively (281, 282). It would be very interesting to determine whether HuD also modulates miRNA-target interactions in β-cells.

#### Competition With Other Non-Coding RNAs

Under specific conditions, endogenous long non-coding RNAs (lncRNAs), pseudogenes, mRNAs, and more recently, circular RNAs (circRNAs) are all capable of interfering with miRNA function by competing with the targets for miRNA binding [reviewed in (283)].

CircRNAs are single-stranded RNAs that form covalently closed loops that arise during splicing (284). Thousands of circRNAs are expressed in pancreatic islets, though the function of only a few has been characterized (241, 285). The effect of a circRNA in target miRNA stability may depend on the cellular context. As briefly mentioned above, Cdr1as (antisense of Cerebellar Degeneration-Related Protein 1, also known as ciRS-7, Circular RNA sponge for miR-7) has been proposed to regulate the expression of miR-7 targets in islets and MIN6 cells. Another circRNA present in human islets, circHIPK3 acts as a sponge of miR-124-3p, miR-338-3p, miR-29b-3p, and miR-30 and is downregulated in islets from db/db mice. Deletion of this circRNA in primary rat β-cells impaired proliferation and promoted apoptosis (241). More recently, high-throughput RNA sequencing was used to identify circRNAs dysregulated in db/db islets. One of the downregulated circRNAs, Circ-Tulp4, was also downregulated by HFD and palmitate, and it has been proposed to control cell proliferation by the interaction with miR-7222-3p (285).

The expression of lncRNAs is also regulated by high glucose, palmitate, and HFD (286). For example, the lnRNA LEGLTBC was downregulated by glucolipotoxicity and functions as an endogenous sponge for miR-34a and a regulator of apoptosis (286). Other examples of miRNAs regulated by lncRNA-sponges are miR-181a-5p, regulated by the plasmacytoma variant translocation 1 (PVT1) lncRNA in INS-1 cells (287) and miR-17, regulated by metastasis-associated lung adenocarcinoma transcript 1 (MALAT1).

Importantly, both lncRNAs and circRNAs often regulate gene expression in a miRNA-independent manner. Regazzi’s lab has made important contributions to the understanding of the function of both lncRNAs and circRNAs in β-cells and have recently generated comprehensive reviews on the subject (288, 289).

#### MiRNA Localization

MiRNA activity can also be modulated by the location and therefore accessibility of the miRNA in and within the cell. Both miRNAs and proteins from the miRISC complex have been found in the nucleus (274, 290), although the functional significance of these miRNAs remains poorly understood. In the nucleus, miRNAs can regulate transcription by promoting chromatin remodelling (291, 292) and have also been suggested to interact with nuclear lncRNAs to modulate their function (293, 294) and to even interact with pri-miRNAs to regulate their processing to mature miRNAs (295). MiRNAs can also be localized into exosomes and represent an important proportion of circulating miRNAs. It is debated whether circulating miRNAs can be merely considered as biomarkers or whether they play regulatory functions. Supporting the latter, increasing numbers of studies demonstrate that cellular-derived exosomal miRNAs can control cellular function and target gene expression in recipient cells, potentially contributing to cross-talk between metabolic organs (296).

The mechanism regulating miRNA localization into exosomes may involve the association with lncRNAs (297), RBPs such as hnRNPA2/B1 or the major vault protein (MVB) (298, 299) and/or Ago proteins (300). Importantly, extracellular miRNAs can also be found free from vesicles, often associated with RBPs or lipoproteins (296, 301, 302). For example, it has been recently shown that β-cells export miR-375 to high-density lipoproteins (HDL) in an specific process tightly regulated by glucose availability, extracellular calcium, and cAMP (303). Several groups have studied changes in the levels of circulating miRNAs in association with hyperglycaemia and/or diabetes. These studies uncovered, for example, a strong association between glycaemia (HbA1c) and elevated circulating miR-125b levels in pre-diabetic (304), T2D (304), and T1D (305) subjects. Moreover, gestational diabetes led to increased serum miR-125b in early pregnancy (306). Our most recent research shows that miR-125b is an abundant islet miRNA regulated by glucose and AMPK (83). Circulating miRNAs may increase as a consequence of damage or alteration of the cells of origin, which in most cases remain to be identified. On the other hand, circulating miRNAs could potentially target β-cells to modulate their function and contribute to the progression of the disease. Pancreatic islets take up and secrete exosomes that affect their own function and that of neighbouring cells (125, 307, 308). For example, lymphocytes infiltrating mouse islets during the development of T1D release exosomal miR-155 that is taken up by β-cells to trigger apoptosis (308). Also, β-cells release miR-29b in response to HFD, which has been proposed to target insulin signalling in the liver (309). On the other hand, the contribution of β-cells to circulating miRNAs (ci-miRNAs) levels has been challenged. For example, only ~1% of the circulating miR-375 originates from β-cells (168), despite being strongly enriched in these cells. Exciting research published in the past few years (310, 311) has identified adipose tissue as an important source of circulating exosomal miRNAs. The liver, skeletal muscle, and perhaps, the gut have also been suggested to release and intake miRNA-containing extracellular vesicles (312). Even though little is known about the destiny and function of ci-miRNAs derived from each of these tissues, considerable effort has been put into identifying the cellular origin of ci-miRNAs. We anticipate that similar studies to those performed by Thomou and colleagues (310), who assessed changes in circulating miRNAs following the elimination of *Dicer* in adipocytes using an adipocyte-specific *Cre* deleter strain, may be useful to ascertain the contribution of pancreatic β-cells, and perhaps other islet cells, to circulating miRNAs.

## Conclusions and Perspectives

Research during the past 15 years has clearly demonstrated that β-cell miRNAs play an essential role in glucose-stimulated insulin secretion and cell survival. It has also been widely demonstrated that diet modulates the expression of islet miRNAs. These nutrient-regulated miRNAs contribute not only to β-cell compensation but also to the β-cell functional failure and death mediated by hyperglycaemia and excess of fatty acids. Nevertheless, the molecular mechanisms leading to these changes in miRNA expression remain mostly unexplored. For example, only a few individual miRNA-transcription factor connections have been studied to some detail. As discussed in detail above, one of the constraints is the lack of accurate annotations of miRNAs transcription start sites. Hopefully, state-of-the-art approaches such as those described above be applied to identify β-cell/islet-specific miRNAs TSS and to study their transcriptional regulation in a high-throughput manner. Moreover, a precise mapping of islet miRNA TSS will also allow us to determine whether Single-Nucleotide Polymorphisms (SNPs) associated with diabetes traits rely within pri-miRNAs or regulatory regions of miRNA genes. This information can be leveraged to identify miRNAs with an important role in the maintenance of glucose homeostasis.

Pancreatic beta cells within the islets have been shown to be heterogeneous at multiple levels (313) and to present a functional and transcriptional heterogeneous response to high-fat diet (314, 315). Whereas single-cell RNA sequencing (scRNA-seq) techniques have evolved considerably during the past few years and have helped to understand β-cell functional heterogeneity, single-cell small RNA-seq has only started to be developed (316). Single-cell miRNA profiling can be used to further our understanding of the molecular mechanisms underlying cellular heterogeneity (317) and to determine whether β-cell changes in miRNA expression in response to nutrients are also heterogeneous.

It is now well established that miRNAs processing and turnover are tightly regulated processes that can have a substantial impact on steady-state cellular miRNA abundance. Moreover, it is now clear that miRNA activity depends not only on miRNA abundance but also on target accessibility. Research in the past recent years has provided novel mechanistic insights into the processes that govern miRNA-target interactions, uncovering important roles for RBPs and other ncRNAs. These regulatory mechanisms play an important part in controlling the function of miRNAs in neurons, which display multiple functional similarities and common regulatory mechanisms of gene expression to β-cells. Nevertheless, the contribution of these pathways to nutrient-mediated changes in β-cell miRNA expression and action remains chiefly unexplored and constitute an exciting new research avenue in the field

Future studies will hopefully fill all these gaps and provide important new findings that will allow us to better understand the contribution of miRNAs to β-cell dysfunction.

## Author Contributions

Both AS and AM-S contributed to the critical review of the literature and drafting of the manuscript. Both authors contributed to the article and approved the submitted version.

## Funding

This work was supported by a New Investigator Research Grant from the Medical Research Council (MRC, MR/P023223/1).

## Conflict of Interest

The authors declare that the research was conducted in the absence of any commercial or financial relationships that could be construed as a potential conflict of interest.

## Publisher’s Note

All claims expressed in this article are solely those of the authors and do not necessarily represent those of their affiliated organizations, or those of the publisher, the editors and the reviewers. Any product that may be evaluated in this article, or claim that may be made by its manufacturer, is not guaranteed or endorsed by the publisher.
